# Supplementary material for: A CATH domain functional family based approach to identify putative cancer driver genes and driver mutations
Source: Sci Rep. 2019 Jan 22;9:263. doi: 10.1038/s41598-018-36401-4 (PMC6343001; doi:10.1038/s41598-018-36401-4)
Supplement: Supplementary file 1 — Supplementary Information [file 41598_2018_36401_MOESM1_ESM.docx]

Supplementary Materials

# A CATH domain functional family based approach to identify putative cancer driver genes and driver mutations

Paul Ashford^1,+^, Camilla S.M. Pang^1,+^, Aurelio A. Moya-García^1,2^, Tolulope Adeyelu^1^ & Christine A. Orengo^1,*^

^+^These authors contributed equally to this work.

^1^University College London, Institute of Structural and Molecular Biology, London, UK.

^2^Laboratorio de Biología Molecular del Cáncer, Centro de Investigaciones Médico-Sanitarias (CIMES), Universidad de Málaga, Málaga, Spain

Correspondence and requests for materials should be addressed to Christine Orengo (email: c.orengo@ucl.ac.uk).

# Supplementary Figures

## Supplementary Figure 1

1. Plot of number of MutFams versus number of tumour samples for 22 cancer types; no correlation is observed.

## Supplementary Figure 1 (continued)

**A**

1. Plot of number of MutFams versus number of mutations for all 22 cancers. A strong correlation is observed when analysing all 22 cancer types (r = 0.84, p < 0.0001). This correlation is largely due to the highly mutated cancers (SKCM, UCEC, COAD, LUAD) having a larger number of MutFams. There is no significant correlation without these highly mutated cancers (r = 0.42, p = 0.08) or when additionally excluding related cancers from the same primary site (i.e. COAD-READ and LUAD-LUSC) (r = 0.38, p = 0.14).

**B**

## Supplementary Figure 2

GO-Slim term enrichment and main cellular processes for uniquely identified genes from MutFam, Miller and consensus datasets.

## Supplementary Figure 3

1.
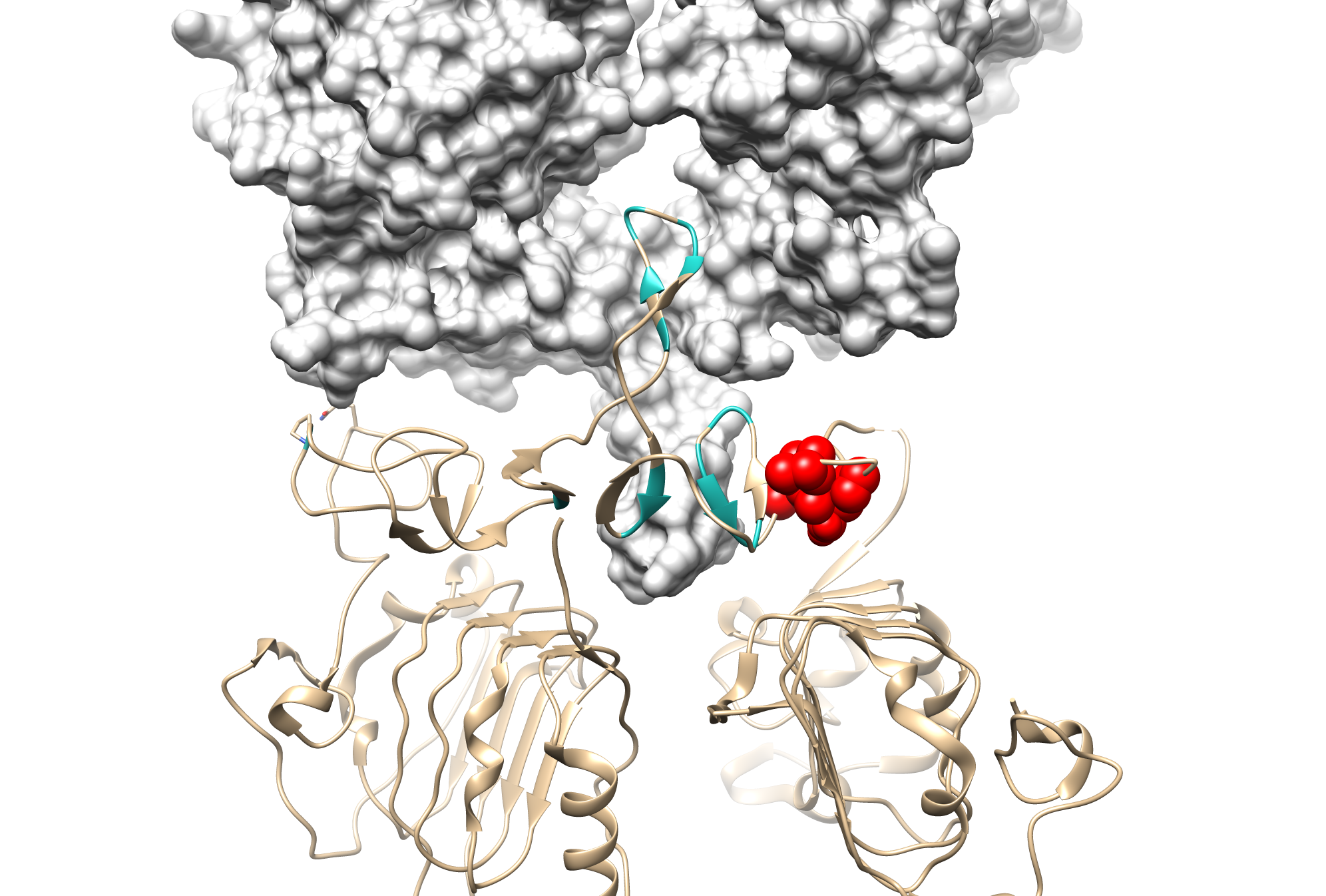
Mutations cluster near the dimerization arm of EGFR (PDB:1mox). Binding of EGF-ligands to EGFR causes dimerization via the extracellular L-domain, causing receptor activation via autophosphorylation in the intracellular kinase domains and downstream growth factor signalling. Shown here are two monomers (grey surface and light brown ribbon) interacting via the extracellular L-domains, which mediate disulphide contacts between EGFR monomers during activation. Key interface residues are marked (cyan ribbon). The clusters (red spheres) are near this interface and suggest a role for these mutations in EGFR activation, which MutFam identified in gliomas, colon adenocarcinomas and lung adenocarcinomas.

A

## Supplementary Figure 3 (continued)

1. Mutation clusters near the catalytic site and ATP pocket of BRAF kinase. BRAF is an oncogene mutated in ~80% of melanomas and in multiple other cancer types, including colorectal, thyroid, ovarian, lung, pancreas and prostate (source CGC). Certain mutations can cause constitutive kinase activation, the most common of which is V600E (shown orange) resulting in increased MAPK pathway activity and cellular growth signaling. We identified mutation clusters near residues 583, 589, 591, 593, 594, 595, 596, 615 [PDB ID: 3ppj] (red spheres) near the ATP binding pocket, where kinase inhibitor FOI is shown bound in this structure. The clusters are also near CSA residues Asp576, Lys578, Asn580, Asn581 and Ser616. We identified MutFams containing BRAF in skin, colorectal, thyroid, lung tumours and gliomas.


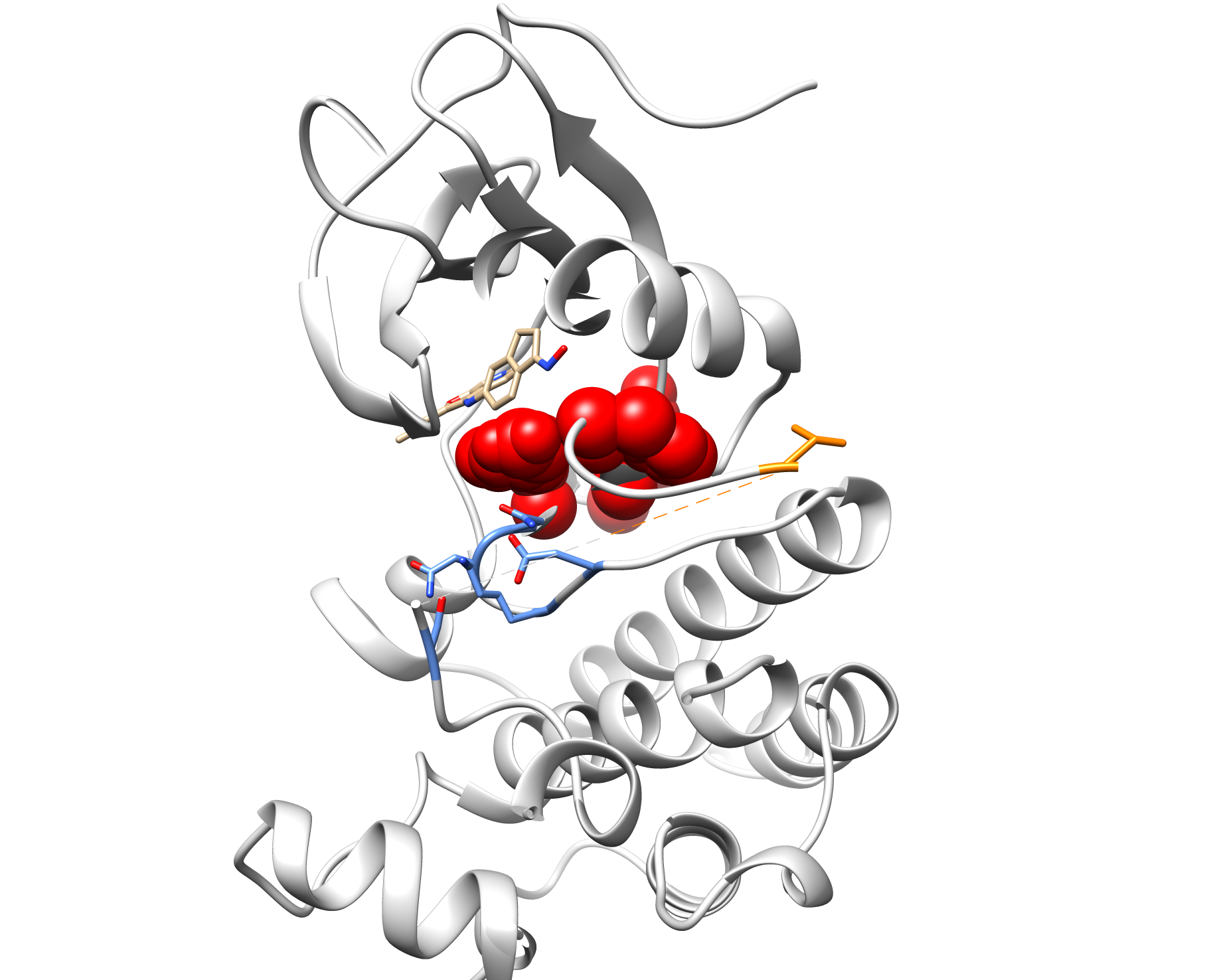


B

**Supplementary Figure 4**

Venn diagram and table summarise top mutated MutFam genes identified from glioma cancer types GLI, LGG and GBM. CGC genes are highlighted in bold.


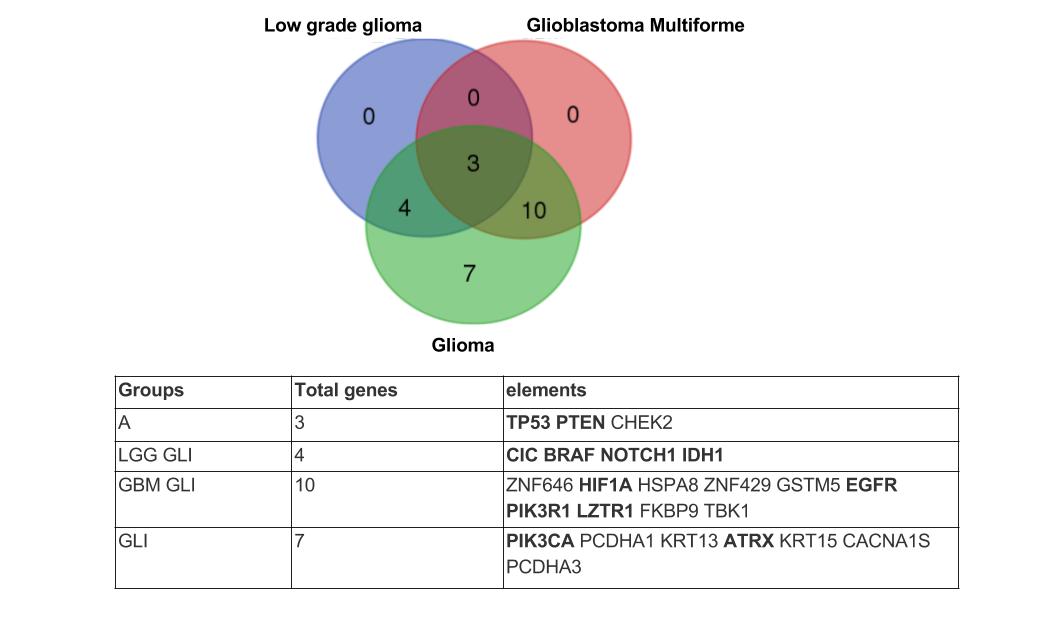


| **Gliomas** | **Number of genes** | **Genes** |
| --- | --- | --- |
| All | 3 | CHEK2, **PTEN**, **TP53** |
| GLI and LGG | 4 | **BRAF, CIC, IDH1, NOTCH1** |
| GLI and GBM | 10 | **EGFR**, FKBP9, GSTM5, **HIF1A**, HSPA8, **LZTR1**, **PIK3R1**, TBK1, ZNF429, ZNF646 |
| GLI only | 7 | **ATRX**, CACNA1S, KRT13, KRT15, PCDHA1, PCDHA3, **PIK3CA** |

# Supplementary Information

## 1.1 Analysis of the gene sets by functional network analysis to identify those more likely to be associated with cancer

##### Biological processes identified for the common driver genes

From the 12 genes common to all datasets we identified 2 functional modules containing 7 genes. Module 1 contained NRAS, KRAS, PIK3CA and RAC1 and was significantly enriched for 9 GO Biological Processes (BPs). Module 2 contained BRAF, FBWX7 and HRAS and was significantly enriched for a single BP (**Supplementary Table 5**). The processes in Module 1 relate to receptor signalling pathways including growth factors (vascular epidermal growth factor, fibroblast growth factor, epidermal growth factor and nerve growth factor) plus insulin signalling. Module 1 also includes immune-related processes such as "Fc-epsilon receptor signalling pathway" and "innate immune response". Module 2 genes were involved in a single signalling-related process, "positive regulation of ERK1 and ERK2 cascade". Both modules involve processes that contribute to known hallmarks of cancer^1^.

##### Common processes in the unique MutFam and Miller modules implicated in Notch signalling

The functional networks derived from the unique MutFam and Miller gene sets both showed modules that were enriched for GO BPs relating to Notch signalling—involved in cellular differentiation—but via contrasting mechanisms (**Supplementary Table 6**).

The MutFam genes in “Notch receptor processing” have mutations in the receptors NOTCH2 and NOTCH4. Genes from the Miller set show enrichment in “Notch signalling pathway” via upstream and downstream regulators of the Notch pathway, such as EP300 (Histone acetylase, involved in chromatin modification), and transcriptional regulators CEBPA, CDK8 and SNAI2. It has been suggested that alterations in cellular development in cancer—implicated by genes found in both the MutFam and Miller sets—play a role in maintaining a pluripotent cellular state that is more adaptable and genetically flexible to changes in the cellular environment and this contributes to cancer cell survival^2^. Therefore, mutations in proteins involved in developmental pathways, such as NOTCH, may act to maintain this pluripotent state and contribute to the tumourigenic phenotype.

##### Common processes in the unique MutFam and Miller modules implicated in DNA binding and transcriptional regulation

Other common process identified in functional modules from the unique MutFam and unique Miller gene sets relate to DNA transcription. Modules from both sets include many different proteins that contain DNA binding zinc-finger domains (**Supplementary Table 7**) implicated in cancer^3^. Mutations to zinc-finger domains may affect the specificity and/or affinity of DNA binding and could therefore compromise DNA quality control, with resulting effects on the cancer hallmark processes of genome stability and regulation^1^. Despite the distinct sets of zinc-finger domain containing genes found in the MutFam and Miller sets, they are enriched in two identical processes: "regulation of transcription, DNA-dependent" and "transcription, DNA-dependent". This result further highlights how distinct driver genes identified by different methods may be acting through alterations to a small number of common biological processes.

##### Cellular development processes identified for the unique MutFam and Miller gene sets

We further analysed distinct functional modules from the unique MutFam and Miller genes that mapped to diverse specific processes that all broadly relate to cellular developmental pathways. For the MutFam genes, we identified three functional modules that were enriched in developmental processes (e.g. “nervous system development”, “axon guidance”, and “synapse assembly” - for a full list see **Supplementary Table 8**). Processes affecting neuronal and system development have been implicated in breast cancer, where changes in neuronal guidance, migratory contacts and ephrin signalling contribute towards carcinogenesis^4,5^. Functional modules identified from the Miller genes are enriched in processes such as “cardiac rhythm” and “regulation of smooth muscle contraction” (for a full list see **Supplementary Table 9**). Manipulation of the cellular cytoskeleton, using contractile elements such as those used in muscle contraction, is a common event in cell migration (specifically the amoeboid mode of migration) enabling invasion through the surrounding extracellular matrix^6^. The process "axon guidance" identified in one module from the Miller set includes RhoGTPases. In the context of cellular development, RhoGTPases are downstream effectors of ephrin receptor signalling, where RhoGTPase activation leads to actin monomer assembly into larger cytoskeletal structures, which is imperative for cellular adhesion and migration - and in turn axon guidance - and is implicated in cancer^7^.

Further analysis showed that whilst the MutFam and Miller gene sets were commonly enriched in biological processes implicated in cellular development, these processes were associated with different gastrulation layers. This is perhaps not surprising as the MutFam and Miller gene sets are associated with different distributions of mutations in cancer types (see **Supplementary Table 10**). We examined any possible tissue bias in the MutFam and Miller studies by comparing them with respect to their tissue origins in gastrulation (**see Supplementary Section 1.2)**. Our results suggest that the MutFam and Miller genes may be involved in similar processes operating in different cellular contexts and this may partly explain why there is a relatively low overlap in the MutFam and Miller gene sets.

## 1.2 Gastrulation layers

The three gastrulation layers - ectoderm, mesoderm and endoderm - are formed during embryogenesis and lead to different tissue types during development. To examine any tissue bias in the MutFam and Miller studies, the cancers analysed by the MutFam and Miller approaches were compared with respect to their tissue origins in gastrulation (**Supplementary Table 10**). Modules identified for the MutFam set tend to be enriched in developmental processes associated with the endoderm layer – which eventually forms the, lung, thyroid, and digestive tract, and the ectoderm layer – which forms the skin epidermis, neurons in the brain, and pigment cells (for a full list of these developmental processes see **Supplementary Table 8**). In contrast, modules for the Miller set (**Supplementary Table 9**) largely involve processes associated with mesoderm layers, which form the cardiac, smooth and skeletal muscle tissues, kidney tubules, and red blood cells. Overall, these findings suggest that the MutFam and Miller genes may be involved in similar processes operating in different cellular contexts and may partly explain why there is a relatively low overlap in the MutFam and Miller gene sets.

## 1.3 Different biological processes identified for the unique MutFam and Miller genes

Biological processes implicated in cancer and uniquely enriched in MutFam modules include “Homophilic cell adhesion” and “calcium dependent cell adhesion” (**Supplemental Table 12**). These modules involve clusters of genes from the Proto-cadherin family, involved in cell adhesion and migration, and are thought to act as a chemical conduit between intracellular signalling and migratory processes during cancer cell invasion^8,9^. The involvement of these processes is further supported by studies that show that ECM reorganisation and alteration of these cell-cell junction proteins give an increased invasion in carcinomas^1,7^. Activation of focal adhesions and the break-down of cell-cell adherences such as cadherin-based Adheren’s junctions, are reminiscent of a phase cancer cells undergo to increase invasion and metastasis – the Epithelial to Mesenchymal Transition (EMT)^1^.

Biological processes unique to modules identified for the Miller set include those implicated in RNA splicing, RNA processing, and gene expression – all of which have shown to be implicated in cancer^10^. Specifically, the affected genes include various Heterogeneous Nuclear Ribonucleoproteins, cyclin dependent kinase 12 (CDK12), and RNA Binding Motifs (RBM), as shown in **Supplementary Table 13**. All genes play a crucial role in facilitating transcription by preventing RNA secondary structures, and aiding mRNA pre-processing and nuclear exportation steps – which have recently been shown to be implicated in tumour progression^11^.

## 1.4 Detailed analysis of MutFam driver genes in brain cancers

Tumours initiating in the glial cells of the brain or spine are termed gliomas. We analysed a set of general gliomas (GLI) and two subtypes, low-grade glioma (LGG) and the more advanced glioblastoma multiforme (GBM). Low-grade gliomas are associated with much better patient prognosis than mature (stage IV) and invasive globlastoma multiforme tumours^12^.

As expected, the two subtypes (LGG and GBM) each have MutFams in common with general gliomas (GLI) (see **Supplementary Figure 4** and **Supplementary Table 2**).

Using the top mutated genes from the MutFams in each of the gliomas, we found that TP53, PTEN and CHEK2 are common to all three cancer types; **Supplementary Figure 4** summarises genes found commonly and uniquely in each glioma type. To interpret how biological pathways containing these genes differed between low-grade gliomas and the more advanced glioblastoma multiforme, we used Functional Interaction Network (FIN) analysis as previously described (see Methods). We were able to map 12 GLI, 8 GBM and 3 LGG genes to each FIN, which we then clustered into functional modules as summarised in **Supplementary Table 14**.

Using GO enrichment, the network module common to GLI, LGG and GBM was enriched in biological processes implicated in DNA repair and cell cycle checkpoint, consistent with tumour suppressive roles of TP53 and CHEK2 in DNA regulation and entry into cell cycle. An additional module found in GBM, but not in LGG, contained genes implicated in later stage glioma processes including immunity and chaperone functions of proteostasis. The GLI dataset, which includes other minor glioma sub-types in addition to the two analysed here, additionally identified cellular adhesion processes.

## 1.5 Supporting evidence for inclusion of genes in Table 1 classified as ‘Other’

Genes in Table 1 (main text) with a “General Function” of “Other” have been analysed below to identify other evidence supporting cancer association for these seven genes.

**TTN**

There are over 4,000 mutations in TTN in the COSMIC pancancer dataset and they are found in all cancer types. High mutation burden likely reflects the very large gene size (>34kbp). However, we only find enrichment in a single cancer type (COAD) in a specific MutFam (enrichment factor = 2.77) indicating that the MutFam method is successfully filtering out most of these mutations. Additionally, TTN has been identified as a potential colorectal cancer associated gene^13,14^.

**SSH1 & SSH3**

There is evidence for SSH1 role in tumour development^15^. SSH1 and SSH3 both contain a protein phosphatase domain.  This domain was identified as a MutFam from multiple mutated genes in colorectal cancers (COAD and READ, genes SSH1, SSH3, DUSP1, DUSP5, DUSP6, DUSP7, DUSP19 & DUSP22) along with a 3D cluster.

**DSP & EPPK1**

Large proteins (DSP ~2.8kbp and EPPK1 ~5kbp) with key role in desmosomes that link neighbouring cells at cell-cell junctions. The pancancer dataset has mutations in these genes in most tumour types.  MutFam identified significant enrichment of the Plectin MutFam only in LUAD, LIHC, SKCM and THCA with additional functional evidence from identified 3D mutation clustering.

**SEC24C**

Functions as part of coat protein complex II (COPII) and identified commonly by MutFam and Miller. A role for SEC24C in cancer via changes to AKT pathway has been suggest^16^.

**CSMD1**

CUB and sushi domain-containing protein 1 is identified commonly by both MutFam and Miller methods and has evidence for a tumour suppressive role in squamous cell carcinomas^17^.

# Supplementary Tables

## Supplementary Table 1

Table summarises number of cancer samples and number of MutFams found by cancer type.

## Supplementary Table 2

Table summarises 405 MutFams grouped into 22 cancer types. Entries grouped by cancer type then ordered by decreasing enrichment factor (which quantifies the over­representation of mutations within the FunFam boundaries across one or more human genes compared with a uniform distribution over whole genes) and corrected *p*-values.

**This table is included as an Excel spreadsheet and online at:**

[**https://paulashford.github.io/mutfam.github.io/**](https://paulashford.github.io/mutfam.github.io/)

## Supplementary Table 3

Table summarises 8 MutFams identified from the polymorphism dataset. Entries ordered by decreasing enrichment factor (which quantifies the over­representation of mutations within the FunFam boundaries across one or more human genes compared with a uniform distribution over whole genes) and corrected *p*-values.

## Supplementary Table 4

Network DS-Scores with associated P-values for MutFam, Miller and combined gene sets. P-values are calculated with respect to randomisation tests (see Methods).

| **DATASET** | **DS-Score** | **P-value (compared to Random)** |
| --- | --- | --- |
| **MutFam** | 1.212 | 1.14 x 10^-13^ |
| **Miller** | 1.463 | 1.00 x 10^-8^ |
| **MutFam & Miller** | 1.314 | 1.12 x 10^-14^ |
| ***Average for 1000 randomisations*** | *1.976* |  |

## Supplementary Table 5

Enriched GO biological processes for network modules identified for common driver genes.

| **Module** | **GO biological processes** | **Gene list** |
| --- | --- | --- |
| 1 | Vascular endothelial growth factor receptor signaling pathway | NRAS, PIK3CA, KRAS, RAC1 |
| 1 | Nerve growth factor receptor signaling pathway | NRAS, PIK3CA, KRAS, RAC1 |
| 1 | Fc-epsilon receptor signaling pathway | NRAS, PIK3CA, KRAS, RAC1 |
| 1 | Blood coagulation | NRAS, PIK3CA, KRAS, RAC1 |
| 1 | Leukocyte migration | NRAS, PIK3CA, KRAS |
| 1 | Fibroblast growth factor receptor signaling pathway | NRAS, PIK3CA, KRAS |
| 1 | Insulin receptor signaling pathway | NRAS, PIK3CA, KRAS |
| 1 | Epidermal growth factor receptor signaling pathway | NRAS, PIK3CA, KRAS |
| 1 | Innate immune response | NRAS, PIK3CA, KRAS, RAC1 |
| 2 | Positive regulation of ERK1 and ERK2 cascade | FBXW7, BRAF, HRAS |

## Supplementary Table 6

Different genes in MutFam and Miller datasets converge via functional modules on Notch receptor signalling.

| **Module** | **GO biological process (MutFam)** | **Gene list** |
| --- | --- | --- |
| 12 | Cell fate determination | NOTCH2, NOTCH4 |
| 12 | Notch receptor processing | NOTCH2, NOTCH4 |
| 12 | Hemopoiesis | NOTCH2, NOTCH4 |
| **Module** | **GO biological process (Miller)** | **Gene list** |
| 1 | Keratinocyte differentiation | TP63, ANXA1, LATS2, EPHA2 |
| 1 | Notch signaling pathway | EP300, TP63, CEBPA, CREBBP, CDK8, SNAI2 |

## Supplementary Table 7

Unique zinc finger DNA binding genes in the Miller and MutFam sets map to network modules, which are associated with common GO biological processes.

| **Module (MutFam)** | **GO biological process** | **Gene list** |
| --- | --- | --- |
| 5 | Regulation of transcription, DNA-dependent | ZNF430, ZNF100, ZNF492, ZNF98, ZNF43, ZNF708, ZNF83, ZNF429, ZNF506, ZNF714, ZNF138, ZNF479, ZNF676, ZNF585B, ZNF267, ZNF431, ZNF585A |
| 5 | Transcription, DNA-dependent | ZNF430, ZNF100, ZNF492, ZNF98, ZNF43, ZNF83, ZNF429, ZNF506, ZNF714, ZNF138, ZNF479, ZNF676, ZNF585B, ZNF267, ZNF431, ZNF585A |
| **Module (Miller)** | **GO biological process** | **Gene list** |
| 1 | Regulation of transcription, DNA-dependent | ZNF28, ZNF572, ZNF571, ZNF250, ZNF470, ZNF208, ZNF527, ZNF845, ZNF768, ZNF286B, ZNF624, ZNF568, ZNF546, ZNF468, ZNF620, ZNF883, ZNF286A, ZNF320, ZNF583, ZNF681, ZNF483, ZNF461, ZNF184, ZNF260, ZFP2, ZNF181, ZNF180, ZNF34, ZNF92, ZNF70, ZNF71, ZNF836, ZNF616, ZNF117, ZNF554 |
| 1 | Transcription, DNA-dependent | ZNF28, ZNF572, ZNF571, ZNF470, ZNF208, ZNF527, ZNF845, ZNF286B, ZNF624, ZNF568, ZNF546, ZNF468, ZNF620, ZNF883, ZNF286A, ZNF320, ZNF540, ZNF583, ZNF681, ZNF483, ZNF184, ZNF260, ZFP2, ZNF181, ZNF34, ZNF92, ZNF70, ZNF71, ZNF836, ZNF616, ZNF117, ZNF554 |

## Supplementary Table 8

Enriched GO processes in network modules identified for MutFam genes in cellular development. Processes also common to Miller highlighted in bold.

| **Module** | **GO Biological process** | **Gene list** |
| --- | --- | --- |
| **1** | Ephrin receptor signaling pathway | ITSN1, KALRN, EPHB1, EPHB3, EPHA7, VAV2, NGEF |
| **1** | **Axon guidance** | ITSN1, KALRN, ROBO1, EPHB1, EPHB3, EPHA7, DCC, UNC5C, UNC5D, VAV2, NGEF |
| **1** | Retinal ganglion cell axon guidance | EPHB1, EPHB3, EPHA7 |
| **1** | Nerve growth factor receptor signaling pathway | ITSN1, KALRN, ARHGEF18, VAV2, NET1, OBSCN, NGEF |
| **1** | Glutamate receptor signaling pathway | GRIK4, GRIK1, GRIK2 |
| **1** | Ionotropic glutamate receptor signaling pathway | GRIK4, GRIK1, GRIK2 |
| **1** | Synaptic transmission, glutamatergic | GRIK4, GRIK1, GRIK2 |
| **1** | Anterior/posterior axon guidance | DCC, UNC5C |
| **1** | Negative regulation of collateral sprouting | EPHA7, DCC |
| **1** | Peptidyl-tyrosine phosphorylation | TTN, EPHB1, EPHB3, EPHA7 |
| **1** | Dendritic spine development | EPHB1, EPHB3 |
| **1** | Regulation of cell-cell adhesion | EPHB3, EPHA7 |
| **1** | Regulation of GTPase activity | EPHB3, VAV2, NGEF |
| **1** | Dendritic spine morphogenesis | EPHB1, EPHB3 |
| **1** | Central nervous system projection neuron axonogenesis | EPHB1, EPHB3 |
| 3 | Nervous system development | PCDHB12, PCDHA1, PCDHB2, PCDHA5, PCDHA4, PCDHA3, PCDHA2, PCDHB3, PCDHA8, PCDHA6 |
| 3 | Synapse assembly | PCDHB14, PCDHB13, PCDHB10, PCDHB2, PCDHB16, PCDHB3 |
| 3 | Synaptic transmission | PCDHB14, PCDHB13, PCDHB10, PCDHB2, PCDHB16, PCDHB3 |
| 14 | Regulation of axon extension involved in axon guidance | PLXNA2, PLXNA1, PLXNA4 |
| 14 | Branchiomotor neuron axon guidance | PLXNA2, PLXNA1, PLXNA4 |
| 14 | Semaphorin-plexin signaling pathway | PLXNA2, PLXNA4 |
| 14 | **Axon guidance** | PLXNA2, PLXNA1, PLXNA4 |
| 14 | Chemorepulsion of branchiomotor axon | PLXNA4 |
| 14 | Cerebellar granule cell precursor tangential migration | PLXNA2 |
| 14 | Postganglionic parasympathetic nervous system development | PLXNA4 |
| 14 | Vagus nerve morphogenesis | PLXNA4 |
| 14 | Anterior commissure morphogenesis | PLXNA4 |
| 14 | Dichotomous subdivision of terminal units involved in salivary gland branching | PLXNA1 |
| 14 | Glossopharyngeal nerve morphogenesis | PLXNA4 |
| 14 | Trigeminal nerve structural organization | PLXNA4 |

## Supplementary Table 9

Enriched GO processes in the network modules identified for the Miller genes in cellular development. Processes common to the MutFam genes are highlighted in bold.

| **Module** | **GO Biological process** | **Gene list** |
| --- | --- | --- |
| 1 | Fat cell differentiation | GSK3B, AKT2, EP300, CEBPA, EGR2 |
| 1 | **Axon guidance** | **GSK3B, PDGFRA, RHOG, RHOB, RASA1, RASA2, EPHA2, RASAL1, RRAS, ERBB4, FGFR1** |
| 1 | Circadian rhythm | GSK3B, DBP, EP300, DYRK1A, NFIL3 |
| 3 | Regulation of smooth muscle contraction | KCNB2 |
| 3 | Locomotor rhythm | KCND2 |

## Supplementary Table 10

Comparison of cancer types within MutFam and Miller genes - the 3 different gastrulation layer columns contain the numbers of the different cancers in each group.

| **Datasets** | **Cancers** | **Ectoderm** | **Mesoderm** | **Endoderm** |
| --- | --- | --- | --- | --- |
| Miller MutFam | SKCM, LIHC, GBM, STAD, UCS LUSC, BLCA, OV, LGG, UCEC, LUAD, COADREAD, THCA, KIRC, LAML, BRCA, PRAD | 3 | 6 | 8 |
| Miller | THCA, LUSC, KICH, OV, KICH, SKCM HNSC, CESC, ACC, UCS, LUAD, KIRP | 2 | 6 | 4 |
| MutFam | DBLC, ESCA, GLI, PAAD | 2 | 1 | 1 |

## Supplementary Table 11

Table summarises 472 genes obtained from the top 25% mutated genes of all 405 MutFams. MutFam codes correspond to CATH Superfamily ID and FunFam number. Columns show MutFam enrichment factor (see Methods), mutations in each gene (within FunFam boundaries) and the evidence supporting gene's involvement in cancer: 3D cluster, GO module, CGC and Miller. A score is calculated by counting the number of supporting evidence types found for each gene. The table is sorted by score, MutFam enrichment factor and total number of mutations in the MutFam (highest to lowest for each). The subset of 151 genes from MutFams with at least one other piece of evidence supporting a role in cancer is obtained by filtering for total score > 0.

**This table is included as an Excel spreadsheet and online at:**

[**https://paulashford.github.io/mutfam.github.io/**](https://paulashford.github.io/mutfam.github.io/)

## Supplementary Table 12

Unique GO biological processes identified in MutFam modules involved in cellular adhesion.

| **Module** | **GO biological process** | **Gene list** |
| --- | --- | --- |
| 3 | Homophilic cell adhesion | PCDHGA6, PCDHGA3, PCDHGA2, PCDHGA1, PCDHB14, PCDHB13, PCDHA13, PCDHB12, CELSR2, PCDHB10, CDH5, PCDHA1, PCDHB2, CDH1, PCDHB16, PCDHA5, PCDHA4, PCDHA3, PCDHA2, PCDHB3, PCDHA9, PCDHA8, PCDHB8, PCDHA6 |
| 3 | Calcium-dependent cell-cell adhesion | PCDHB14, PCDHB13, PCDHB10, PCDHB2, PCDHB16, PCDHB3 |
| 3 | Cell adhesion | PCDHB12, PCDHA1, PCDHB2, PCDHA5, PCDHA4, PCDHA3, PCDHA2, PCDHB3, PCDHA8, PCDHA6 |

## Supplementary Table 13

Unique GO biological processes identified in MutFam modules involved in RNA splicing.

| **Module** | **GO biological process** | **Gene list** |
| --- | --- | --- |
| 2 | RNA splicing | HNRNPA3, HNRNPH1, HNRNPF, HNRNPH2, CDK12, RBM5 |
| 2 | Nuclear mRNA splicing, via spliceosome | HNRNPA3, HNRNPH1, HNRNPF, HNRNPH2, RBM5 |
| 2 | Regulation of RNA splicing | HNRNPH1, HNRNPF, CDK12 |
| 2 | Gene expression | HNRNPA3, HNRNPH1, HNRNPF, MAPKAPK2, HNRNPH2, RBM5 |
| 2 | RNA processing | HNRNPH1, HNRNPF, RBM5 |

## Supplementary Table 14

Functional network module genes identified in gliomas.

| **Cancer** | **Module** | **Nodes in modules** | **Node list** |
| --- | --- | --- | --- |
| LGG | 1 | 3 | CHEK2, PTEN, TP53 |
| GBM | 1 | 5 | CHEK2, HIF1A, PIK3R1, PTEN, TP53 |
| GBM | 2 | 3 | EGFR, HSPA8, TBK1 |
| GLI | 1 | 6 | HIF1A, NOTCH1, PIK3CA, PIK3R1, PTEN, TP53 |
| GLI | 2 | 4 | CHEK2, EGFR, HSPA8, TBK1 |
| GLI | 3 | 2 | PCDHA1, PCDHA3 |

1. Hanahan, D. & Weinberg, R. A. Hallmarks of Cancer: The Next Generation. *Cell* **144,** 646–674 (2011).

2. Giachino, C. *et al.* A Tumor Suppressor Function for Notch Signaling in Forebrain Tumor Subtypes. *Cancer Cell* **28,** 730–742 (2015).

3. Jen, J. & Wang, Y.-C. Zinc finger proteins in cancer progression. *Journal of Biomedical Science* **23,** 53 (2016).

4. Harburg, G. C. & Hinck, L. Navigating breast cancer: axon guidance molecules as breast cancer tumor suppressors and oncogenes. *J Mammary Gland Biol Neoplasia* **16,** 257–270 (2011).

5. Nasarre, P., Potiron, V., Drabkin, H. & Roche, J. Guidance molecules in lung cancer. *Cell Adhesion & Migration* **4,** 130–145 (2014).

6. Paňková, K., Rösel, D., Novotný, M. & Brábek, J. The molecular mechanisms of transition between mesenchymal and amoeboid invasiveness in tumor cells. *Cell. Mol. Life Sci.* **67,** 63–71 (2010).

7. Sanz-Moreno, V. & Marshall, C. J. The plasticity of cytoskeletal dynamics underlying neoplastic cell migration. *Current Opinion in Cell Biology* **22,** 690–696 (2010).

8. Hulpiau, P. & van Roy, F. Molecular evolution of the cadherin superfamily. *Int. J. Biochem. Cell Biol.* **41,** 349–369 (2009).

9. Berx, G. & van Roy, F. Involvement of members of the cadherin superfamily in cancer. *Cold Spring Harb Perspect Biol* **1,** a003129–a003129 (2009).

10. Piñero, J., Berenstein, A., Gonzalez-Perez, A., Chernomoretz, A. & Furlong, L. I. Uncovering disease mechanisms through network biology in the era of Next Generation Sequencing. *Scientific Reports* **6,** 24570 (2016).

11. Tien, J. F. *et al.* CDK12 regulates alternative last exon mRNA splicing and promotes breast cancer cell invasion. *Nucleic Acids Res* **45,** 6698–6716 (2017).

12. Walid, M. S. Prognostic factors for long-term survival after glioblastoma. *The Permanente journal* **12,** 45–48 (2008).

13. de Voer, R. M. *et al.* Identification of Novel Candidate Genes for Early-Onset Colorectal Cancer Susceptibility. *PLoS genetics* **12,** e1005880–19 (2016).

14. Vilar, E. & Tabernero, J. Molecular dissection of microsatellite instable colorectal cancer. *Cancer discovery* **3,** 502–511 (2013).

15. Maimaiti, Y. *et al.* SSH1 expression is associated with gastric cancer progression and predicts a poor prognosis. 1–7 (2018). doi:10.1186/s12876-018-0739-5

16. Sharpe, L. J., Luu, W. & Brown, A. J. Akt Phosphorylates Sec24: New Clues into the Regulation of ER-to-Golgi Trafficking. *Traffic* **12,** 19–27 (2010).

17. Ma, C. *et al.* Characterization CSMD1 in a large set of primary lung, head and neck, breast and skin cancer tissues. *Cancer Biol. Ther.* **8,** 907–916 (2009).
